# Fossilized Venom: The Unusually Conserved Venom Profiles of *Heloderma* Species (Beaded Lizards and Gila Monsters)

**DOI:** 10.3390/toxins6123582

**Published:** 2014-12-22

**Authors:** Ivan Koludarov, Timothy N. W. Jackson, Kartik Sunagar, Amanda Nouwens, Iwan Hendrikx, Bryan G. Fry

**Affiliations:** 1 Venom Evolution Lab, School of Biological Sciences, the University of Queensland, St. Lucia, Queensland 4072, Australia; E-Mails: jcoludar@gmail.com (I.K.); tnwjackson@gmail.com (T.N.W.J.); iwanhx@yahoo.com (I.H.); 2 Institute for Molecular Bioscience, the University of Queensland, St. Lucia, Queensland 4072, Australia; 3 Department of Ecology, Evolution and Behavior, the Alexander Silberman Institute for Life Sciences, Hebrew University of Jerusalem, Jerusalem 91904, Israel; E-Mail: anaturalist@gmail.com; 4 School of Chemistry and Molecular Biosciences, University of Queensland, St. Lucia, Queensland 4072, Australia; E-Mail: a.nouwens@uq.edu.au

**Keywords:** adaptive evolution, venom, toxin, Heloderma, beaded lizard, gila monster

## Abstract

Research into snake venoms has revealed extensive variation at all taxonomic levels. Lizard venoms, however, have received scant research attention in general, and no studies of intraclade variation in lizard venom composition have been attempted to date. Despite their iconic status and proven usefulness in drug design and discovery, highly venomous helodermatid lizards (gila monsters and beaded lizards) have remained neglected by toxinological research. Proteomic comparisons of venoms of three helodermatid lizards in this study has unravelled an unusual similarity in venom-composition, despite the long evolutionary time (~30 million years) separating *H. suspectum* from the other two species included in this study (*H. exasperatum* and *H. horridum*). Moreover, several genes encoding the major helodermatid toxins appeared to be extremely well-conserved under the influence of negative selection (but with these results regarded as preliminary due to the scarcity of available sequences). While the feeding ecologies of all species of helodermatid lizard are broadly similar, there are significant morphological differences between species, which impact upon relative niche occupation.

## 1. Introduction

There are five extant species of helodermatid lizards: *Heloderma alvarezi*, *H. charlesbogerti*, *H. exasperatum*, *H. horridum and H. suspectum* [1,2]. *H. suspectum* last shared a common ancestor with the other extant species approximately 30 million years ago. *H. exasperatum* and *H. horridum*, the other two species included in this study, last shared a common ancestor approximately 4 million years ago. All are native to the south-western part of the North American continent and inhabit rocky, semiarid and scrubland habitats. Such an absence of appreciable intraclade diversity in ecology is reflected in the overall extreme similarity in morphology of the species. These lizards are also characterised by having a very low metabolic rate and are known to exhibit a preference for low-body temperatures, spending most of the year at temperatures lower than 25 °C [3]. *Helodermatid* lizards raid the nests of birds and rodents [4] but may also predate upon adult rodents [5].

The genus *Heloderma* has been recognised as venomous for more than a century. The teeth are deeply grooved and the glands are very large. Most cases of human envenomation involve lizards biting and holding with their strong jaws, sometimes for hours. Envenomations by helodermatid lizards may be clinically complex, with symptoms including extreme pain, acute local swelling, nausea, fever, faintness, myocardial infarction, tachycardia, hypotension, and inhibition of blood coagulation [6,7,8,9,10,11]. Studies of helodermatid lizard venom have identified several components (Table 1). Of these, exendin-4, isolated from *Heloderma suspectum* venom, is a peptide agonist of the glucagon-like peptide (GLP) receptor that promotes insulin secretion. It has been clinically used to treat type 2 diabetes and to enhance plasma insulin secretion [12].

Variation in venom profiles has been extensively documented between snake species of the same genus [13,14,15,16,17,18,19,20,21,22,23,24,25] and between individuals of the same species, with intraspecific differences found among different geographic localities [16,26,27,28,29,30,31], and between juveniles and adults [28,32,33,34]. Such taxonomic, geographic and ontogenetic variation has been linked to strong natural selection in response to differing prey species [17,28,31,35,36,37,38,39,40,41].

Traditionally, reptile venom research has focused mostly on clinically important snake species. As a result, our understanding of the evolution of helodermatid lizard venom is limited. In this study, we compare the venom proteomes of *H. exasperatum*, *H. horridum* and *H. suspectum* in order to gauge the extent of the diversification in venom composition that has occurred over 30 million years since these species last shared a common ancestor [1].

**Table 1:** *Heloderma* venom peptides/proteins which have been proteomically characterized.

Protein Type/ Toxin Class	Toxic Action	Uniprot Accession #(s)
CRiSP (cysteine rich secretory protein)	Paralysis of peripheral smooth muscle and induction of hypothermia through blockage of various channels including ryanodine and L-type calcium channels.	Q91055
Exendin	Induces hypotension via relaxation of cardiac smooth muscle.	C6EVG1, C6EVG2, P04203, P04204, P20394, P26349
Helofensin	Lethal toxin that inhibits direct electrical stimulation of the isolated hemi-diaphragm.	C6EVG6, D2X5W3, D2X5W4, Q7LZ31
Kallikrein	Increase of vascular permeability, production of hypotension, stimulation of inflammation in addition to cleavage of fibrinogen.	P43685, C6EVG4, C6EVG5
B-type Natriuretic peptide/helokinestatin precursor	Natriuretic peptides produce hypotension through the relaxation of aortic smooth muscle. The helokinestatin peptides are antagonists of bradykinin at the B2 bradykinin receptor.	C6EVG7, D7FB56, D7FB57, E8ZCG5
Phospholipase A_2_ (Type III)	Inhibition of platelet aggregation via the epinephrine-induced pathway.	C6EVG9, C6EVH0

## 2. Results and Discussion

Shotgun sequencing recovered toxin types previously known from the Heloderma venom proteome: CRiSP, exendin, kallikrein, helokinestatin and Type III phospholipase A_2_ (Table S1). In addition, this analytical technique recovered types previously known only from transcriptome studies: hyaluronidase, natriuretic peptide and nerve growth factor.

**Figure 1:**
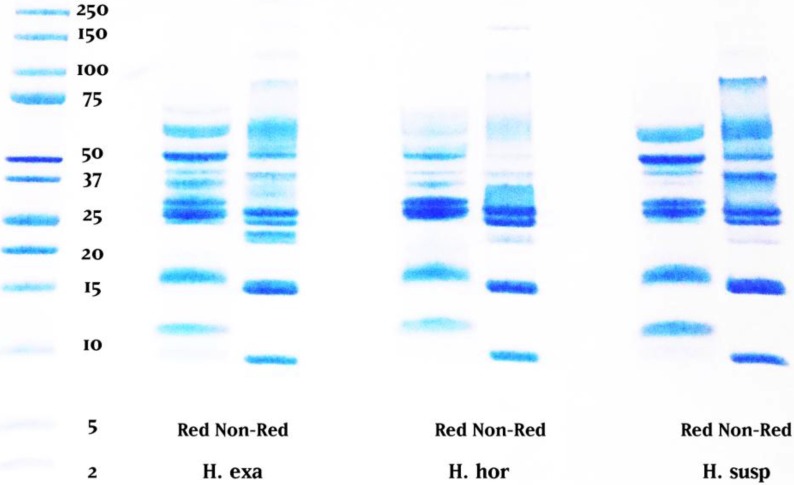
Reduced and non-reduced tris-tricine 1D-gel comparison of *H. exasperatum*, *H. horridum* and *H. suspectum*.

One-dimensional gel electrophoresis (1D-GE) utilizing the tris-tricine method indicated a gross overall similarity between the three venoms (Figure 1). For each species there was a notable difference between non-reduced (NR) and reduced (R) samples. Most notably, a 100 kDa band was present in the non-reduced lanes but absent from the reduced lanes. Conversely, the reduced lanes exhibited a dark band at 50 kDa not present in the non-reduced, indicating that the 100 kDa band was a disulfide-linked dimer.

**Figure 2:**
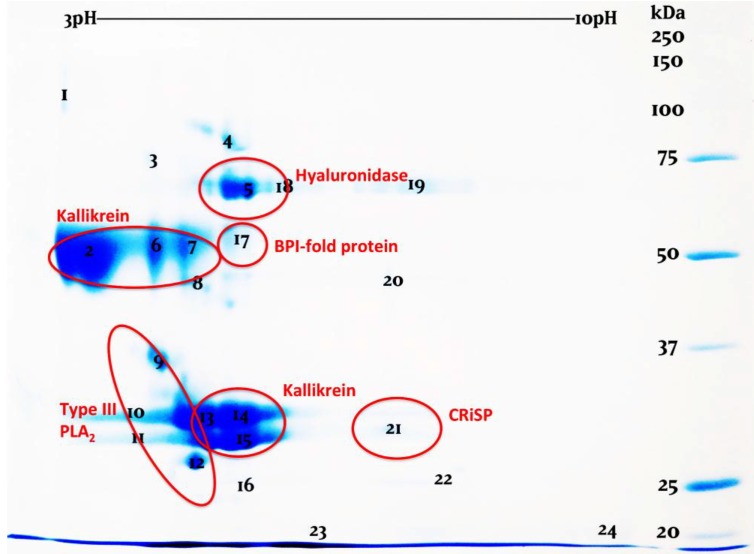
2D-gel examination of *H. exasperatum* venom.

**Figure 3:**
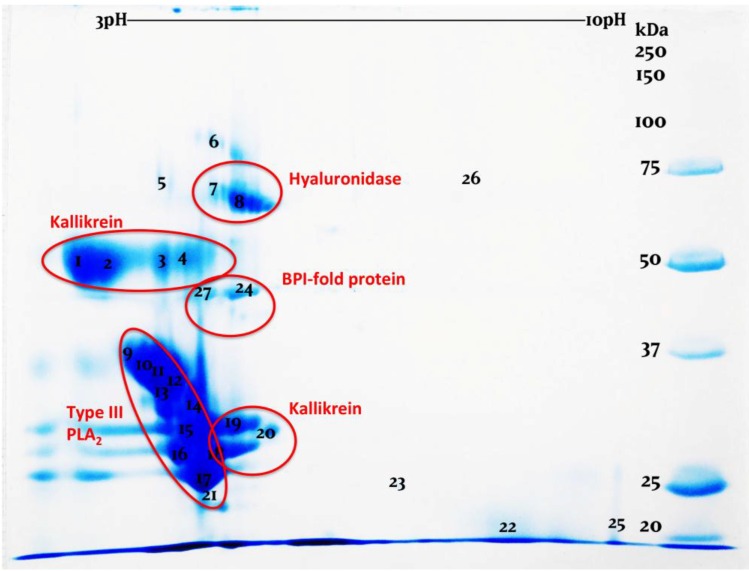
2D-gel examination of *H. horridum* venom.

**Figure 4:**
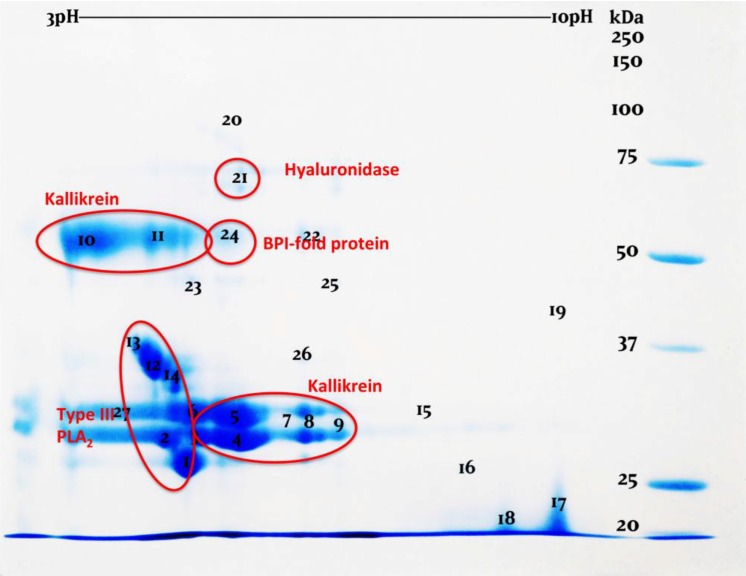
2D-gel examination of *H. suspectum* venom.

2D gels (2D-GE) confirmed the strikingly similar nature compositions of the three venoms (Figure 2, Figure 3 and Figure 4, Table S2). While the venoms are broadly similar in overall protein composition, it is clear that there are significant differences in relative expression levels. This was most apparent in the PLA_2_ region. While these variations may point towards differential evolution, they may be also the result of intergel variation or simply arbitrary variation in venom gland content between individuals at the time of milking. More extensive comparative sampling is required to elucidate individual variation *vs.* species level variations. Regardless, the overall protein composition was vastly more conserved than has been noted even for closely related species of snake (*cf.* [42]).

**Figure 5:**
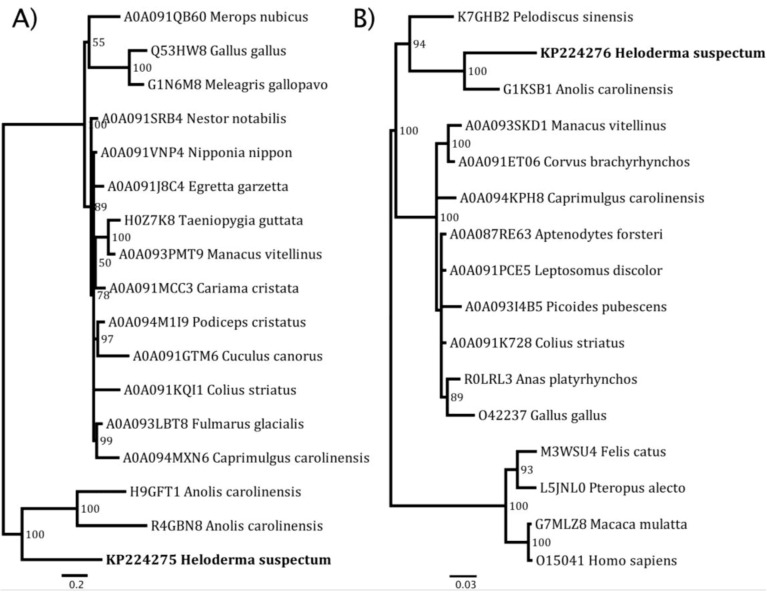
Phylogenetic reconstruction of (**A**) BPI-fold and (**B**) semaphorin proteins. Previously known sequences are referred to by their uniprot accession codes while *Heloderma suspectum* sequences obtained in this study are referred to by their genbank codes.

**Figure 6:**
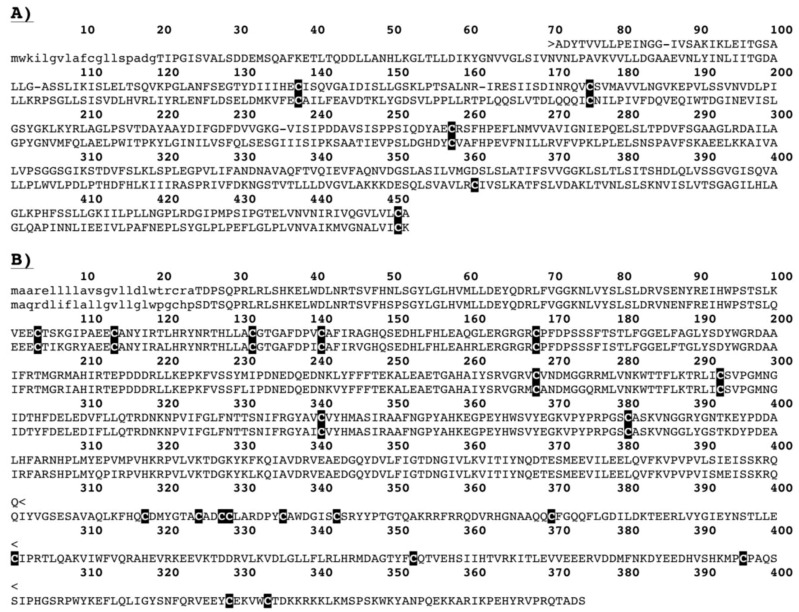
Sequence alignment of (**A**) the BPI-fold proteins from *Heloderma suspectum* venom (KP224275) and *Anolis caronlinensis* genome (R4GBN8) and (**B**) the semaphorin proteins from *Heloderma suspectum* venom (KP224276) and *Anolis caronlinensis* genome (G1KSB1).

2D-GE revealed in all venoms previously unknown venom components which were identified as bactericidal/permeability-increasing (BPI)-fold (all species) and semaphorin proteins by searching the LC-MS/MS results against our previously constructed *H. suspectum* venom gland cDNA library [43,44]. While semaphorin was identified in *H. suspectum* only, it must be noted that light spots located in similar regions of the *H. exasperatum* and *H. horridum* gels, for which mass spectrometry analysis was unable to provide an identity, suggest that this component is likely present in the other *Heloderma* venoms. mRNA sequences are given in Supplemental File 1 and genbank accession numbers are KP224275 (BPI-fold) and KP224276 (semaphorin). Phylogenetic analysis for both protein types identified *Anolis* genome sequences as the nearest known relative (Figure 5 and Figure 6). The fact that both of these were identified as being transcribed by the venom gland indicates that they are indeed secreted by this gland and are not mucus contaminants. Therefore, they may play an as yet unidentified role in envenomation.

The precise role of venom in the ecology of helodermatid lizards remains unknown. Beck [45] considered it a “paradox” that helodermatid lizards hold on “with bulldog tenacity” when biting in apparent defense, thus increasing the lizard’s chance of injury or death. However, this assertion merely reflects a common fallacy of evolutionary thinking—that the individual lizard is the “unit of selection”. As selection takes place at the level of the gene, the death of the individual lizard does not preclude strong selection for “bulldog tenacity” in defensive bites, as presumably this tactic maximises the unpleasantness of the encounter for the aggressor, thus ensuring it avoids such encounters in future. However, the overall pharmacological profile of the venom includes components with lethal neurological effects or other severe physiological targeting, actions not consistent with a purely defensive role for the venom as defensive toxins are typically pain inducing [36]. Rather such lethal effects point towards the venoms having at least some role in predation.

Very few sequences are available and most are from *H. suspectum* and thus selection analyses must therefore be regarded as extremely preliminary. Regardless, pairwise-estimation of omega (non-synonymous (dN) to synonymous (dS) ratio) in this study revealed a greater influence of negative selection on the major toxin-encoding genes of *Heloderma* lineage (Table 2), even for comparisons between *H. horridum* and *H. suspectum* sequences. The absence of diversity in venom-composition and toxin-encoding genes within the sister *Heloderma* lineages suggest that the venoms are not evolving under the diversifying selection pressure characteristic of the predator-prey “chemical arms races” in which venomous organisms utilising their venom for prey subjugation find themselves [31,35,36,41]. However, as noted above, there are obvious variations in relative expression levels within toxin classes and thus these relative expression levels may be a novel form of diversification.

While the venom has actions consistent with predatory use, a defensive venom role is also supported by the aposematic colouration of helodermatid lizards, the fact that these lizards are slow moving and vulnerable above ground (the osteoderms in their skin are another line of defense against would-be predators), and the fact that the lizards often feed on “defenseless” prey such as eggs and nestlings [3]. That being said, the results of the present study do not refute the hypothesis that helodermatid lizard venom may be used (at least partially) for predation, as all species occupy similar ecological niches and therefore feed on similar prey items. It must be emphasised that some toxins have actions consistent with predatory effects including lethal effects upon blood pressure, coagulation and neurological function. More in-depth analyses of the venom gland transcriptomes of helodermatid lizards to mine “enough” nucleotide sequences for evolutionary selection analyses may shed light in this regard.

**Table 2:** Relative toxin molecular evolutionary rates.

Toxin Type	Sequence pairs	Estimates
Kallikrein	EU790962.1 (*H. suspectum*) *vs.* HM437246.1 (*H. horridum*)	dN: 0.180; dS: 0.225; dN/dS: 0.80
	EU790963.1 (*H. suspectum*) *vs.* HM437246.1 (*H. horridum*)	dN: 0.242; dS: 0.450; dN/dS: 0.53
	EU790962.1 (*H. suspectum*) *vs.* EU790963.1 (*H. suspectum*)	dN: 0.081; dS: 0.173; dN/dS: 0.47
	Average	dN: 0.167; dS: 0.282; dN/dS: 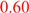
CRiSP	EU790958.1 (*H. suspectum*) *vs.* U13619.1 (*H. horridum*)	dN: 0.011; dS: 0.022; dN/dS: 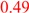
Helofensin	GQ918270.1 (*H. suspectum*) *vs.* EU790964.1 (*H. suspectum*)	dN: 0.030; dS: 0.036; dN/dS: 0.84
	GQ918271.1 (*H. suspectum*) *vs.* EU790964.1 (*H. suspectum*)	dN: 0.052; dS: 0.065; dN/dS: 0.80
	GQ918271.1 (*H. suspectum*) *vs.* GQ918270.1 (*H. suspectum*)	dN: 0.020; dS: 0.027; dN/dS: 0.74
	Average	dN: 0.034; dS: 0.042; dN/dS: 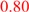

## 3. Experimental Section

### 3.1. Venom Collection

Venoms were obtained from captive bred adult male specimens of *Heloderma exasperatum* (Rio Fuerte, Mexico founder stock), *Heloderma horridum* (Colima, Mexico founder stock) and *Heloderma suspectum* (Phoenix, Arizona, USA founder stock).

### 3.2. Shotgun Sequencing

In order to identify low molecular weight peptides that do not resolve well on 1D or 2D gels, shotgun sequencing was used. Three µg of crude venom sample was dissolved in 50 µL of 100 mM ammonium carbonate to reduce and alkylate cysteine bonds with subsequent addition of 50 µL of 2% iodoethanol/0.5% triethylphosphine in acetonitrile. The sample was afterwards resuspended in 20 µL of 40 mM ammonium bicarbonate, before overnight incubation (at 37 °C) with 750 ng of sequencing grade trypsin (Sigma-Aldrich, Castle Hill, Australia). To stop digestion, 1 µL of concentrated formic acid was added to each of the samples. Samples were lyophilised then resuspended in 20 µL of 5% ACN/0.5% FA, put into MS vials and subjected to LC-MS/MS analysis.

### 3.3. One-Dimensional Gel Electrophoresis

In order to compare venom proteomes between species, 1D gradient gels were run under both reducing and non-reducing conditions using the manufacturer (BioRad, Brisbane, Australia) protocol. Gels were prepared as follows: 0.05 mL Milli-Q H2O, 2.5 mL 30% acrylamide mix, 1.5 mL 1.0 M Tris-HCl, pH 8.45, 0.48 glycerol, 20 µL 10% APS, 2 µL TEMED (spreading gel); 0.76 mL Milli-Q H_2_O, 0.76. mL 30% acrylamide mix, 0.76 mL 1.0 M Tris-HCl, pH 8.45, 15 µL 10% APS, 2 µL TEMED (spacer gel); 1.56 mL Milli-Q H_2_O, 0.34 mL 30% acrylamide mix, 0.63 mL 1.0 M Tris-HCl, pH 8.45, 15 µL 10% APS, 2 µL TEMED (stacking gel). Spreading gel was cast first. After it set, the spacer gel was slowly layered atop it, and after the spacer gel set the stacking gel was layered atop it. Running buffers were: 0.2 M Tris-HCl, pH 8.9 (anode buffer); 0.1 M Tris-tricine-HCl pH 8.45. The gels were run at 100 V for three hours at room temperature. Thirty µg of venom was reconstituted in Tricine loading buffer (Bio-Rad, Brisbane, Australia) with 10 mM DTT added to provide reduce conditions. Gels were stained overnight with colloidal Coomassie brilliant blue G250 (34% methanol, 3% phosphoric acid, 170 g/L ammonium sulphate, 1 g/L Coomassie blue G250). After the staining was complete, gels were destained using ultrapure water (PURELAB Flex 2, Brisbane, Australia).

### 3.4. Two-Dimensional Gel Electrophoresis

In order to further investigate the proteomics variation, particularly that of isoelectric variation, 2D gels were run. Then, 0.3 mg of venom sample were solubilized in 125 µL of rehydration buffer (8 M urea, 100 mM DTT, 4% CHAPS, and 0.5% ampholytes (Biolytes pH 3–10, Bio-Rad, Brisbane, Australia)) with 0.01% bromophenol blue. The sample was mixed with shaking and centrifuged for 5 min at 4 °C, 14,000 rpm. This was done to remove any insoluble material. The supernatant was loaded onto IEF strips (ReadyStrip, non-linear pH 3–10, 7 cm IPG) (Bio-Rad, Brisbane, Australia) and left overnight for passive rehydration. Protein focusing was achieved via PROTEAN i12 IEF CELL (Bio-Rad, Brisbane, Australia). The IEF running conditions were as follows: 100 V for 1 h, 500 V for 1 h, 1000 V for 1 h and 8000 V until 98,400 V/h. Actual current in the final step of the run varied in accordance to resistance. To each strip a constant current of 50 µA was applied. After the run IPG strips were incubated for 10 min in a reducing equilibration buffer (50 mM Tris-HCl, pH 8.8, 6 M urea, 2% SDS, 30% glycerol, 2% DTT) to reduce cysteine bonds. To alkylate reduced bonds IPG strips were further incubated for 20 min in an alkylating equilibration buffer (50 mM Tris-HCl, pH 8.8, 6 M urea, 2% SDS, 30% glycerol, 2.5% iodoacetamide). After rinsing with SDS-PAGE running buffer, IPG strips were positioned on top of 12% polyacrylamide gels (Protean-II Plus, 18 × 20 cm, Bio-Rad Lab, Brisbane, Australia) using 0.5% agarose. Gels were run with a current of 10 mA/per gel for 20 min followed by 20 mA/per gel for the rest of the run until the bromophenol dye front was within 0.5 cm of the base of the gel. After the run, gels were briefly washed with water and stained with 0.2% colloidal Coomassie brilliant blue G250 overnight. Water was used to remove the excess of the dye after staining was complete. Visible spots were subsequently picked from gels and digested overnight at 37 °C with the use of sequencing grade trypsin (Sigma-Aldrich, Brisbane, Australia). Afterwards gel spots were washed with MiliQ water, destained (40 mM NH_4_CO_3_/50% acetonitrile (ACN)) and dehydrated (100% ACN); rehydration occurs in 10 µL of 20 µg/mL TPCK trypsin with subsequent incubation at 37 °C overnight. To elute peptides following solutions were used per each spot: 20 µL of 1% formic acid (FA), followed by 20 µL of 5% ACN/0.5% FA. Collected peptides were put into MS vials and subjected to LC-MS/MS analysis.

### 3.5. LC-MS/MS

In order to identify the toxin types present, digested gel spots and digested whole venom (shotgun) samples were processed using an Agilent (Brisbane, Australia) Zorbax stable bond C18 column (2.1 mm by 100 mm, 1.8 µm particle size, 300 Å pore size) at a flow rate of 400 µL per minute and a gradient of 1%–40% solvent B (90% acetonitrile, 0.1% formic acid) in 0.1% formic acid over 15 min or 4 min for shotgun samples and 2D-gel spots, respectively, on a Shimadzu (Brisbane, Australia) Nexera UHPLC coupled with an AB SCIEX (Brisbane, Australia) 5600 Triple TOF mass spectrometer. MS2 spectra are acquired at a rate of 20 scans per second with a cycle time of 2.3 s and optimised for high resolution. Precursor ions were selected between 80 and 1800 *m*/*z* with a charge state of 2–5 and of an intensity of at least 120 counts per second with a precursor selection window of 1.5 Da. The isotopes within 2 Da were excluded for MS2. MS2 spectra were searched against known translated transcriptome libraries or UniProt database with Proteinpilot v4.0 (ABSciex, Brisbane, Australia) using a thorough identification search, specifying iodoacetamide as an alkylation method, trypsin digestion and allowing for biological and chemical modifications (ethanolyl C or deamidated N in particular) and amino acid substitutions, including artefacts induced by the preparation or analysis processes. This was done to maximize the identification of protein sequences. Spectra were inspected manually to eliminate false positives.

### 3.6. Pairwise-Estimation of dN/dS

Pairwise-estimates of dN/dS were obtained for *Heloderma* Kallikreins, CRiSPs and lethal toxins using the Codeml program of PAML package.

## 4. Conclusions

Due to the limited amount of previously available proteomic data, these results significantly contribute to our understanding of helodermatid lizard venoms. Despite the *H. suspectum* having been separated from all other extant species for 30 million years, the venoms have a significant overall level of similarity in regards to protein/peptide types present but with variable expression within these conserved classes. This suggesting that their venoms experienced a diversifying selection pressure different from that often governs the evolution of venom in other squamate reptiles such as snakes which display significant differences in the types of proteins expressed, even at low taxonomical levels. This variation pattern is likely a consequence of the fact that all helodermatid lizards intrinsically occupy the same ecological niche and thus feed upon the similar prey items. However, the discovery of novel components represents an exciting opportunity for biodiscovery and reinforces the basic premise that poorly investigated venomous lineages represent untapped resources of molecules with potential for utilization in drug design and development.

## Supplementary Materials

Supplementary materials can be accessed at: http://www.mdpi.com/2072-6651/6/12/3582/s1.
